# Genetically Engineered Peptides for Inorganics: Study of an Unconstrained Bacterial Display Technology and Bulk Aluminum Alloy

**DOI:** 10.1002/adma.201301646

**Published:** 2013-07-19

**Authors:** Bryn L Adams, Amethist S Finch, Margaret M Hurley, Deborah A Sarkes, Dimitra N Stratis-Cullum

**Affiliations:** U.S. Army Research LaboratoryRDRL-SEE-B, 2800 Powder Mill Road, Adelphi, MD 20783, USA; Goldbelt RavenLLC, 10 N Jefferson St, Frederick, MD 21701, USA; US Army Research LaboratoryRDRL-WML-B, 4600 Deer Creek Loop, Aberdeen Proving Ground, MD 21005, USA

Biological systems have evolved the exquisite ability to spatially combine many weak, non-covalent chemical interactions to direct the molecular recognition and self-assembly of incredibly complex materials. The ability to control assembly at the molecular level has led to an interest in harnessing nature’s building blocks (e.g., polypeptides, DNA, etc.) to bind inorganic or synthetic compounds for multi-scale fabrication (nano-to macro) of advanced materials. The utility of this approach is evidenced by the large and growing body of research reports highlighting peptides generated through biopanning of surface display peptide libraries.^1^–^5^ Examples include a wide range of peptide binders to pure metals,^6^–^10^ metal oxides,^11^–^13^ metal alloys,^14^ metal salts,^15^ and semiconductors,^16^–^18^ as well as hydroxyapatite—the inorganic component of teeth and bone.^19^ Inorganic binding peptides, no matter the source, are widely recognized for their specificity and design control, and present a remarkable opportunity for advanced materials development.^20^ However, the rules governing this type of peptide binding are not fully understood.^18^, ^20^, ^21^ A variety of factors have been implicated in playing a role in peptide-inorganic surface interactions, including conformational effects,^22^–^25^ electrostatic effects,^26^, ^27^ relative residue placement in the sequence,^28^, ^29^ acid-base chemistry,^30^ and hydrogen bond formation.^14^, ^21^

Discovery of genetically engineered peptides for inorganics (GEPI) through biopanning surface display peptides is most commonly accomplished using phage display technology.^4^ The mainstream use of phage display is due in large part to the commercial availability of M13 bacteriophage display libraries, the diversity of the libraries, and the robustness of the viral host to shear-forces encountered in some biopanning methods. Despite the lack of current commercial availability, bacterial systems, including a number of different *Escherichia coli* (*E. coli*) display technologies (e.g., FliTrx bacterial flagellar display) have been utilized for the discovery of GEPI including inorganic metal-binding peptides.^31^ One key advantage of a bacterial system is that the cells and corresponding genetic material of *E. coli* are relatively easy to manipulate, allowing customized libraries to be generated and transformed at very high efficiencies. *E. coli* also has a very rapid growth rate and is easy to culture, which makes biodiscovery of novel peptides a relatively simple process. In contrast to phage-display, the peptide sequences are directly encoded in the bacterial DNA, resulting in a self-sustaining and replicating population that can easily be propagated without requiring elution from the target, thereby minimizing loss of the peptides possessing the greatest interaction.

Recently, an *E. coli* peptide display library has been developed that offers the greatest estimated diversity (3 × 10^10^ discreet random peptides) to date and is comparable to phage display peptide diversity estimates.^32^, ^33^ A unique feature of this library is the display of unconstrained peptides (15mers) on an engineered outer membrane protein scaffold, eCPX. The unconstrained nature of the peptide is of particular importance because the utility of other bacterial peptide libraries has been limited due to poor accessibility to the cell surface, low sequence diversity, and host cell toxicity effects.^34^ The eCPX peptide library has shown great potential recently in biopanning for affinity peptide binders for protein targets in a rapid (less than one week), semi-automated biopanning method.^35^, ^36^

In this report, we demonstrate for the first time the development of a methodology for *E. coli* peptide discovery to bulk, inorganic targets using an unconstrained bacterial display peptide library. Using this method, a new series of peptides were identified and their binding interactions characterized. We chose to investigate a readily available aluminum alloy as the initial target and demonstrate the versatility of this display scaffold by incorporating programmed peptides, including aluminum binding peptides produced by phage display.^14^ Computational simulation and analysis of peptide conformational fluctuations were used to increase our understanding of sequence-dependent, structure-function relationships. Furthermore, we are the first to show that these relationships contribute to high affinity peptide interactions with this aluminum system.

In order to develop bulk aluminum binding peptides from an *E. coli* eCPX peptide display library, a new biopanning methodology was first developed (**Figure 1**A). Biopanning is an affinity-based selection technique in which high affinity peptide binders are enriched from a peptide library containing millions to billions of individual, genetically encoded cells each displaying a unique peptide sequence. Isolation and amplification of the peptide isolates with the highest affinity is accomplished through several steps: (1) binding (to immobilize the peptide materials with the greatest affinity to the target material), (2) washing with a series of stringency pressures put on the system (to remove unbound or weekly bound peptides), and (3) enrichment though regrowth of the remaining library members (to build up the population of peptides with the desired properties). The process is repeated several times (typically 3-5 rounds), to enrich the target population with the desired binding properties, and the stringency conditions employed are critical to a successful enrichment process. Negative selections can also be used against materials with similar properties to improve target specificity. Due to the fact that the combinatorial display library is genetically encoded, identification of the final population is easily accomplished through standard DNA sequencing techniques.

**Figure 1:**
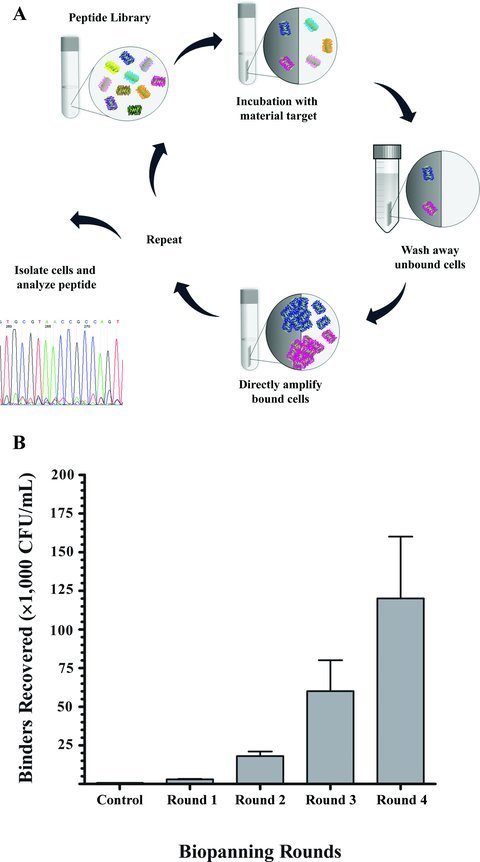
A) Schematic diagram of the biopanning process developed for discovery of metal binding peptides to bulk aluminum using an unconstrained bacterial peptide display library. B) Experimental results showing enrichment of aluminum isolates through progressive rounds of biopanning.

In order to monitor the enrichment, an indirect binding assay was developed to recover the population from the aluminum surface after each round, and these results are shown in Figure 1B. Although the indirect assay is not quantitative, a substantial increase in the relative number of bound cells from each successive round was observed, with an overall 40-fold increase from the fourth (and final) round relative to the first. The negative control was identical to any other cell in the library except for the lack of the unique 15mer peptide. It is important to note that few to no cells were recovered from the negative control samples, indicating that the appropriate stringency was employed during the biopanning process. Also, negligible binding by the negative control demonstrates that binding was most likely facilitated by the displayed peptides, and that neither general bacterial cell adhesion elements nor the display scaffold itself had a significant contribution to aluminum surface binding.

Analysis through DNA sequencing of isolated round 4 colonies revealed 17 unique sequences (**Table 1**). All but one sequence exhibited the full length (15mer) peptide, with the exception of DBAD10 (12mer). This truncation was not due to a stop codon or frameshift, and similarly, truncated peptides have been previously isolated from the parent library in other studies with protein target systems.^35^ It is important to note that while the peptide is truncated in DBAD10, a full length eCPX scaffold was verified via sequence analysis and expression levels were monitored during FACS analysis. The peptide isolate designated DBAD5 was present once in the population sampled and exhibited the greatest number of hydroxyl and sulfoxyl containing residues. However, the isolated peptide sequence designated DBAD1 was the only sequence present more than once (identified 49 times), and possessed seven hydroxyl and sulfoxyl containing residues. To investigate this further, the 17 isolated colonies (i.e., isolated peptide binders) were assessed individually for their relative affinity to the aluminum alloy (**Figure 2**A) using the indirect binding assay. Overall, the relative affinity to the aluminum target varied significantly, spanning a 2-log variation. All isolates exhibited a greater interaction with the aluminum target, compared to the negative control, and the peptide isolate DBAD1 had a marked increase in interaction, relative to all other peptides. Specifically, DBAD1 exhibited a 360-fold higher target binding relative to the lowest isolated binder (DBAD14), and significantly higher than the next best isolate (DBAD24). It is important to note that the number and composition of isolates were not influenced by a competitive growth advantage. A comparison of planktonic growth (doubling time) of DBAD1 expressing cells to other isolates under the growth and induction conditions utilized during biopanning discovery is provided in Supplemental Figure 1.

**Figure 2:**
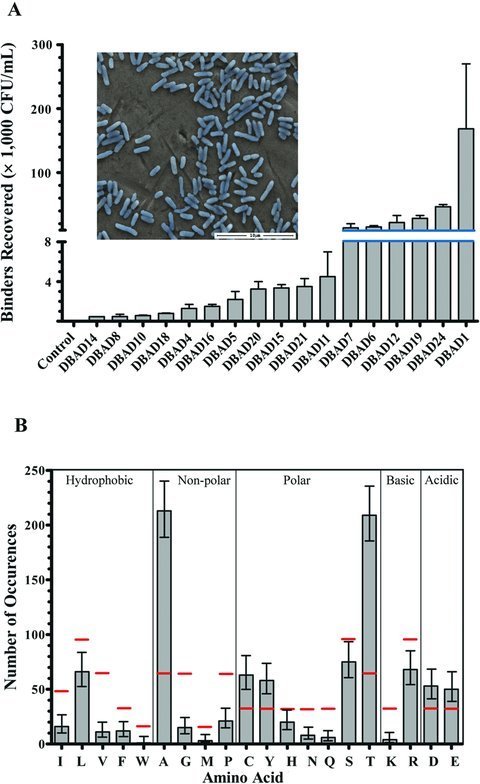
Analysis of aluminum binding peptides. A) Comparison all 17 peptide isolates using an indirect binding assay with aluminum. Inset shows a representative scanning electron microscopy (SEM) image of the DBAD1 isolate bound to the bulk aluminum alloy. B) Statistical analysis allowing comparison of observed and expected frequency of amino acid residues across all 17 peptide isolates. Gray bars indicate the observed residue occurrences in each peptide and corresponding 95% confidence interval. Red lines indicate the theoretical expected residue occurrences, assuming the library was fully randomized.

**Table 1:** Aluminum peptide sequences isolated from round 4 biopanning population with notation of frequency of occurrence and number of hydroxyl or sulfoxyl groups in each peptide. Hydroxyl and sulfoxyl residues are underlined.

Name	Peptide Sequence	Number Hydroxyl or Sulfoxyl Groups
DBAD1	S T E A R A T T L T A C D A Y	7
DBAD5	L F H R S C P S Y D T Y S C L	8
DBAD4	H I G P S R Y S S A F H C L S	6
DBAD10	S S C C S I H H R D C F	6
DBAD7	G S M F I L T G F T G T V S H	5
DBAD19	D H C F R I P N L P T Y R S C	5
DBAD8	Q V H P R G S Y H R A P S I C	4
DBAD11	A S R T A L R C V Q H R V R T	4
DBAD14	N G A T I C K A H P S A L V T	4
DBAD16	K Y R P C Y P R L K P F I H T	4
DBAD6	S N I A P I P R N H F I H T S	3
DBAD12	P Q A L N S Y S A I F A A I N	3
DBAD15	V N V S Y A W F V H G S R R M	3
DBAD18	S T V Q A F G P G C V A Q H L	3
DBAD20	S G H H C D K E I G A R L L H	2
DBAD21	V S P P G P H L R G A L P I G	1
DBAD24	L P R I P G N L F T I L Q P M	1

To further verify binding interaction from the peptide isolates, scanning electron microscopy (SEM) was used to directly visualize DBAD1 binding to bulk aluminum (a representative figure shown in the Figure 2B inset). Supplemental Figure 2 provides a side-by-side comparison of DBAD1 to the negative control after 24 h of incubation followed by stringent removal of unbound cells. Overall, from these data it can be concluded that (1) the biopanning method was successful against a bulk aluminum material, (2) the displayed peptide strongly facilitates the interaction of the isolates with the aluminum target, and (3) the DBAD1 isolate exhibited significantly better binding performance, warranting further investigation.

Although peptide-metal and peptide-metal oxide interactions (e.g., Cu_2_O, ZnO, GaAs crystals, TiO_2,_ etc.) are not fully understood, the consensus of research in this area indicates that the peptide isolates are categorized by a predominance of polar, hydroxyl-containing residues with little to no positional consensus across the isolated population.^7^, ^14^, ^16^, ^26^, ^37^ The exact mechanism of the interaction of hydroxyl-containing residues with the aluminum oxide (alumina) surface remains open to debate.^14^ However, it is feasible that some measure of hydrogen bonding similar to that found in solvent/oxide surface interactions^38^–^40^ and peptide/oxide interactions^27^ may exist. When considering the peptide isolates reported herein (Table 1), it is noteworthy that the hydroxyl containing residues are distributed throughout the length of the majority of these peptides—a fact we will investigate in detail. However, it should also be noted that two of the sequences contain only a single hydroxyl-containing residue, implying that multiple mechanisms for surface binding must exist. A more thorough analysis of the exact mechanism of these interactions is left to future work.

The combined residues from the 17 isolates in Table 1 (weighted by frequency) were examined further for trends (enrichment or depletion) in amino acid character using a similar analysis to that employed by Thai et al.^26^ Figure 2B summarizes these calculated results into general categories of hydrophobic, non-polar, polar, basic, and acidic. The experimental frequency of each residue is displayed in the bar graph, along with the corresponding 95% confidence interval. The theoretical residue frequency in a given sequence can be estimated from the 20 possible naturally occurring amino acids. However, the DNA codons used to encode the amino acids displayed causes some residues to be present at a higher frequency (i.e., degeneracy) than others. Accordingly, the red lines in Figure 2B represent the theoretical expected residue occurrences, assuming the library was fully randomized. As expected, polar residues, the prime candidate for surface interactions, were enriched. While basic residues were under-represented, the charged acidic residues (glutamic and aspartic acid) were elevated in a moderate but statistically relevant fashion. Previous analysis of poly-L-glutamic acid on an aluminum oxide surface has demonstrated the dependence of the interaction on pH and salt concentration, positing the direct role of the carboxylate group in binding.^41^ Through computational modeling, Dringen et al. demonstrated the adsorption of glutathione disulfide (GSSG, g-GluCysGly disulfide) on alumina nanoparticles and also indicated direct involvement of the carboxylate groups.^30^ The statistically large increase in alanine, however, is unexpected. As the side chain of alanine is a simple methyl group, it is unlikely that this enrichment is due to a direct chemical interaction. It is more likely that structural considerations govern these interactions, since alanine is noteworthy as a helix-forming residue.

Similar to our studies, Zuo et al.^14^ developed peptides with affinity to aluminum and steel alloys. Their work also suggested that aluminum binding peptides have an expected bias toward hydroxyl-containing amino acids. The DBAD1 peptide isolate has seven hydroxyl containing residues distributed over a peptide length of 15 residues. In comparison Al-S1, the highest affinity peptide in the work by Zuo, has five hydroxyl containing residues distributed over a peptide length of 12 residues. It is difficult to directly compare binding affinities of peptides of varying length, developed and displayed on different scaffolds.^1^ However, to further demonstrate the versatility of the eCPX cell surface display scaffold, and directly compare the relative performance with an aluminum binding peptide developed by phage, we genetically engineered the system to allow programmed display of peptide sequences, including Al-S1. Due to the unconstrained peptide display scaffold of eCPX, peptides of dissimilar length from different scaffold origins can be readily incorporated. Comparison of Al-S1 to DBAD1 using the indirect binding assay performed in Figure 2A indicated successful binding by Al-S1 displayed on the eCPX scaffold to the bulk aluminum alloy with significantly greater recovery of DBAD1 (Supplemental **Figure 3**). Although the indirect assay and analysis is not quantitative, the versatility of the eCPX scaffold with the ability to compare and translate peptides derived from different sources to a biofilm producing system is demonstrated.

**Figure 3:**
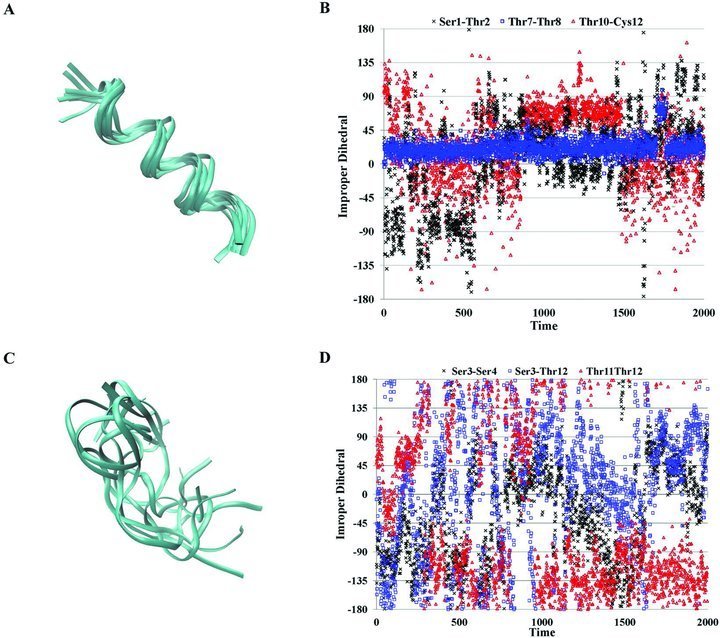
Molecular dynamics study of DBAD1 and A1-S1. (A) and (C) show an overlay of peptide backbone during simulation trajectory for the DBAD1 and Al-S1 peptides, respectively. (B) and (D) show the behavior of improper dihedrals marking relative orientation of hydroxyl groups during the course of simulation for the DBAD1 and Al-S1 peptides, respectively.

The prominence of helix-forming alanine residues in DBAD1 and helix-breaking proline residues in Al-S1 led us to perform molecular dynamics simulations to study structural characteristics that may facilitate surface binding. Figures 3A and 3C shows an overlay of each peptide backbone structure during the course of a 40 ns simulation trajectory for peptides DBAD1 and Al-S1, respectively. Al-S1 rapidly lost the initial helical structure and maintained mostly turn and random coil secondary structure (helicity 0.4%). By contrast, DBAD1 maintained the initial helical core structure very well (helicity 83%). This agrees with previous studies, which have noted the stability of helices in short (16 residue) alanine-based peptides.^42^ Therefore, while both DBAD1 and Al-S1 had a relatively high number of hydroxyl and sulfoxyl groups to present to the aluminum surface, the manner in which these groups were presented to the surface differed.

This can be demonstrated through an analysis of the relative orientation of successive hydroxyl-(or sulfoxyl-) containing side chains along the length of each peptide. To quantify this relative orientation, we measured an improper dihedral angle (Φ_ij_), defined by the hydroxyl oxygens (or sulfoxyl sulfur) and backbone carbons of two (not necessarily adjacent) residues, i and j. A visual representation of this moiety is shown in Supplemental Figure 4. A Φ_ij_ value in the range of ± 90° implies rough alignment along the same face of the peptide, and denotes the possibility for multiple, simultaneous binding sites as the peptide approaches the surface. A measure of this is shown in Figures 3B and 3D, where the improper dihedral angle (Φ_ij_) has been tabulated for various pairs of hydroxyl-containing residues along the peptide length during the course of the simulation for DBAD1 and Al-S1, respectively. A marked contrast in the behavior for these two peptides is very evident. DBAD1 maintained a helical character and presented multiple aligned binding groups all along the peptide length. This alignment is shown for residue pairs Ser 1-Thr2, Thr8-Thr10, and Thr10-Cys12, representing a range of locations and lengths scales within the peptide. While some scatter outside the range of ± 90° is seen, the residue alignment overwhelmingly fluctuates quite tightly about 0. In contrast, Al-S1 lacked an overarching structure and any potential binding groups were scattered over the perimeter of the peptide (shown for residue pairs Ser3-Ser4, Ser3-Thr12, and Thr11-Thr12). It is obvious from this analysis that pairs of hydroxyl groups in the Al-S1 peptide had little to no alignment with each other. This means that in the absence of an overriding helical structure, the availability of an individual residue for binding as the peptide approaches the surface is largely a matter of chance. Both peptides will bind, but we suggest that this structural behavior strongly contributes to the improved binding affinity of DBAD1 relative to Al-S1. Further analysis is currently underway on additional peptides, specifically looking for alternative conformational and binding group alignment modes to enable us to draw more general conclusions on features that contribute to successful binding interactions.

To conclude, we demonstrated for the first time a novel and general approach to GEPI discovery using an unconstrained *E. coli* peptide library. Using this approach, we have discovered a unique DBAD1 peptide isolate that we believe to have superior binding performance with the aluminum (alumina) alloy, and attribute the increased interaction to a propensity to sustain an overarching helical structure with preferential presentation of hydroxyl-and sulfoxyl-containing residues to the metal surface. Compared to conventional techniques, our methodology enables direct propagation of the isolated material throughout the GEPI discovery process. This allows for the selection of the best affinity isolates, which are often lost in competing methodologies requiring elution at extreme pH conditions from the bulk material during the biopanning process. The computationally-driven methods employed, allow for a greater understanding of potential structure-function relationships and offer a new standard for GEPI analysis extending to other discovery systems (e.g., phage, yeast, etc.). We believe due to the demonstrated simplicity and versatility, our general methods will broadly extend current capabilities of GEPI discovery towards bulk, inorganic and more complex materials. Furthermore, our work is likely to have significant impact to the design and development of beneficial biofilms, including living paint for common metals (including aluminum) subject to corrosion. Future studies will continue to explore the use of this biological tool for advanced material development and improved understanding of hybrid material interactions.

## Experimental Section

*Bacterial Strains, Culture Conditions, and Materials*: In all biopanning experiments, a previously developed *E. coli* unconstrained peptide display library constructed from an eCPX display scaffold was utilized.^32^, ^33^, ^43^ These materials were obtained from the laboratory of Dr. Patrick Daugherty (University of California Santa Barbara) and cells were cultivated and maintained as previously described.^35^ Phage derived aluminum binding peptide^14^ Al-S1 (VPSSGPQDTRTT) was synthesized (BioBasics) for cloning into a eCPX vector using standard molecular biology methods. Primers used to amplify the peptide insert for cloning were as follows: Forward 5′-TTCCGTAGCTTGTACATGTGGCCAG-3′ and Reverse ′-CACCGCTGCCACCGCT-3′. The 83 bp insert was ligated into the empty display vector, pBad33-nl3, which was constructed with BsrGI and XhoI digestion sites for peptide sequence cloning. The resulting plasmid, named pBad33-AB1, was then transformed into chemically competent MC1061 cells and insertion of the programmed peptide sequence verified by sequencing (Genewiz).

Samples of aluminum sheet (product 5052-H32 Aluminum Sheet, onlinemetals.com) were received as 0.16 cm thick, and 5 cm × 10 cm in size. Prior to use, the aluminum was cut into a size (1 cm × 5 cm) compatible with standard culture tubes and samples were autoclaved using standard sterilization cycle. All molecular and microbiology support materials (e.g., primers, buffers, enzymes, media, Tween20, antibiotics, etc.) were obtained from standard, commercial suppliers (Fisher Scientific, Sigma Aldrich, Invitrogen, NEB, etc.) and used according to standard techniques.

*Biopanning Method*: Prior to biopanning against the target, the eCPX bacterial display library was prepared as previously described, with arabinose (0.04%) induction occurring at an OD_600_ 0.50–0.55 for 35–45 min.^35^ After induction, the cells were chilled on ice for 15–30 min. Sterilized aluminum samples were added to the induced library and placed on a shaker at 4 °C for 15 min. The aluminum samples were briefly rinsed in sterile phosphate buffered saline (PBS) and transferred to PBS supplemented with Tween20 (1%) (PBST). The samples were washed for 5 min and stringency of isolation wash adjusted through additional wash steps with each successive round of biopanning to remove loosely bound cells. After washing, bound cells were recovered by removing the aluminum samples to LB+Cm/Glu and growing at 37 °C with shaking overnight. This overnight culture was then used in the subsequent round, for a total of 4 rounds. Ninety-six randomly selected colonies from round 4 were sequenced using the pBAD Forward universal primer (Genewiz) and the peptides identified from the generated sequences using the InsertMultiSeek analysis tool (www.sequencetools.com).

*Indirect Binding Assay*: The aluminum binding propensity of each sorting round, a population consisting of a single isolate or the empty display vector (negative control) were compared by quantifying the number of cells recovered from the aluminum surface. This assay was carried out by initially diluting overnight cultures 1:100 into fresh LB+Cm (5 mL), followed by eCPX expression induction with arabinose as described previously. The induced cells were then chilled on ice for 15-30 min before addition of sterile aluminum samples for 15 min at 37 °C with shaking. The aluminum samples were briefly rinsed in sterile PBS and transferred to PBST (30 mL) and shaken at 150 rpm at room temperature for 30 min, a simpler washing regime that was found to yield the same results as the most stringent regime used during biopanning (data not shown). The aluminum samples were removed to LB+Cm/Glu (6 mL) and incubated at 37 °C with shaking for 1 hour. This incubation step allowed bound cells to be replicated off the aluminum surface. Furthermore, the addition of glucose prevented the expression of the eCPX display scaffold and thus, the cells remained planktonic and could then be enumerated. This was performed by serial dilutions on LB+Cm agar plates and the number of cells mL^−1^ recovered from the aluminum surface tabulated. All samples were prepared as duplicate independent samples, the results averaged, and the standard error of the means calculated.

*Planktonic Growth Study*: The isometric growth of the isolates were measured by constructing growth curves of the negative control (i.e., empty display vector) and cells displaying peptides Al-S1, DABD1, DBAD24, DBAD8, and DBAD14 in either LB+Cm, LB+Cm with an arabinose induction described previously, or in LB+Cm/Glu. Additionally, a growth curve was also constructed in the LB+Cm/Glu recovery condition for these strains following a typical arabinose induction and 45 min incubation as described previously using a 1:100 dilution. All samples were prepared as duplicate independent samples and the doubling time and 95% confidence intervals were calculated using the nonlinear fit exponential growth equation (Prism 5, GraphPad Software).

*Scanning Electron Microscopy*: Cell binding to an aluminum alloy surface was directly visualized microscopically. An overnight culture of MC1061 cells harboring either the pBad33-DBAD1 or pBad33-nl3 plasmids were diluted 1:100 in fresh LB+Cm (50 mL) and induced with arabinose, as described previously. After induction, aluminum SEM stubs were added directly to the culture and incubated at 37 °C for 24 hours before transfer to dI H_2_O (30 mL) and shaken at 150 rpm at room temperature for 15 min. Samples were then removed and allowed to air dry prior to imaging using a FEI Quanta 200FEG ESEM (FEI) scanning electron microscope. A comparison of cell binding to the aluminum SEM stubs and the bulk aluminum alloy used in binder development were found to be similar (data not shown) and use of the stubs did not require additional sample preparation, as the use of the bulk aluminum did, and yielded better quality, uncoated images.

*Statistical Analysis of Critical Amino Acids*: Statistical analysis was used to compare the observed number of occurrences of each amino acid found to expected number of occurrences of each amino acid based on codon degeneracy. Although there was a 17 unique peptide sequences found, a total of 65 individual sequences were analyzed because sequence DBAD1 was identified 49 times. Statistical significance of differences between the observed and expected values were determined by calculating the 95% confidence intervals for each residue, as described by Thai et al.,^26^ using the MAPLE software.^44^ Briefly, the probability of any residue’s occurrence is governed by binomial distribution. By using the cumulative binomial probability function (described in detail in reference^26^), which accounts for the number of sequences isolated and the peptide length, the upper and lower 95% confidence limit can be calculated. The values are displayed as error bars in Figure 2B and are asymmetric due to the use of a binomial distribution.

*Molecular Dynamics and Helicity Simulations*: Individual molecular dynamics simulations were performed for each peptide of interest. Peptide structures were built within VMD^45^ from sequence information, solvated with water, and sufficient ions added to neutralize the system. The system was then minimized for 5000 steps, heated to 300 K, and NPT dynamics performed for approximately 40 ns. The simulations were performed using the CHARMm forcefield with a timestep of 2 fs and pressure of 1 atm with the NAMD software of Schulten et al.^46^ The propensity for alpha helix formation of peptides were calculated utilizing the scale of Pace and Schultz.^47^ STRIDE analysis^48^ of the DBAD1 and Al-S1 secondary structure show an average percent helicity of 83% and 0.4%, respectively over a 40 ns simulation.

## Supporting Information

As a service to our authors and readers, this journal provides supporting information supplied by the authors. Such materials are peer reviewed and may be re-organized for online delivery, but are not copy-edited or typeset. Technical support issues arising from supporting information (other than missing files) should be addressed to the authors.
