# Supplementary material for: Genetically Engineered Peptides for Inorganics: Study of an Unconstrained Bacterial Display Technology and Bulk Aluminum Alloy
Source: Adv Mater. 2013 Jul 19;25(33):4585–91. doi: 10.1002/adma.201301646 (PMC3793233; doi:10.1002/adma.201301646)
Supplement: Supplementary file 1 [file adma0025-4585-sd1.pdf]

Copyright WILEY-VCH Verlag GmbH & Co. KGaA, 69469 Weinheim, Germany, 2013.

ADVANCED MATERIALS

Supporting Information

for *Adv. Mater.*, DOI: 10.1002/adma.201301646

# Genetically Engineered Peptides for Inorganics: Study of an Unconstrained Bacterial Display Technology and Bulk Aluminum Alloy

*Bryn L. Adams , Amethist S. Finch , Margaret M. Hurley , Deborah A. Sarkes , and Dimitra N. Stratis-Cullum **

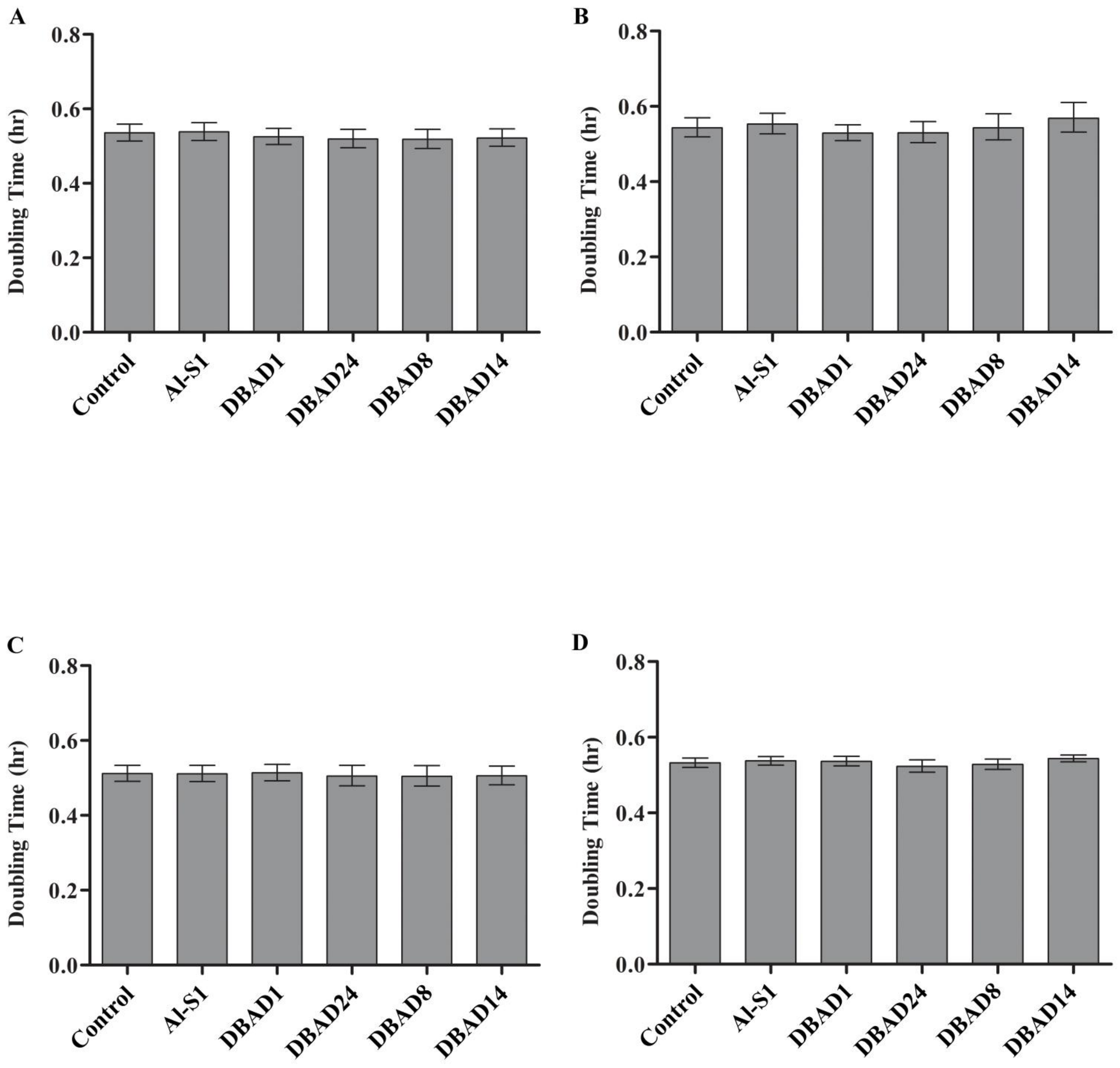


**Supplemental Figure 1.** Comparison of cell growth (doubling time) for the negative control eCPX, the engineered, phage-derived aluminum binding peptide (Al-S1), the best two aluminum binding peptides (DBAD1 and DBAD24) and the poorest two aluminum binding peptides (DBAD8 and DBAD14), in the following growth conditions: A) LB+Cm, B) LB+Cm with a 0.04% arabinose induction at $OD_{600}$ 05-0.55, C) LB+Cm and 0.2% glucose, D) 1:100 dilution into LB+Cm and 0.2% glucose after growth in LB+Cm with a 0.04% arabinose induction at $OD_{600}$ 05-0.55 and a 45 min inducation.

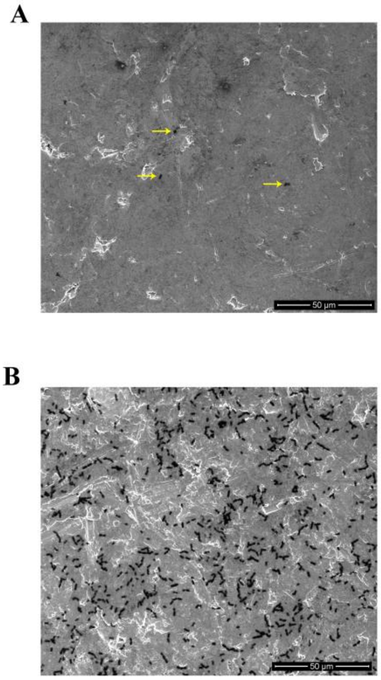


**Supplemental Figure 2.** SEM of *E. Coli* bound to bulk aluminum after 24 hour incubation and stringent washing (A) Negative control (eCPX empty display vector) with bound cells denoted by yellow arrows. (B) DBAD1 isolate bound cells.

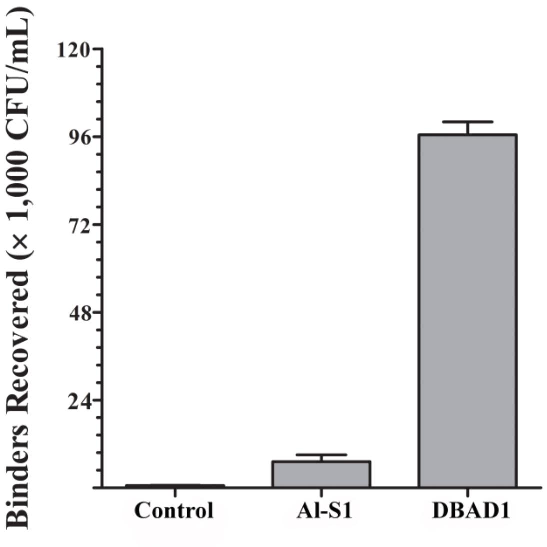


**Supplemental Figure 3.** Comparison of aluminum binding with bacterial derived DBAD1 and phage derived Al-S1 displayed on the eCPX display scaffold.

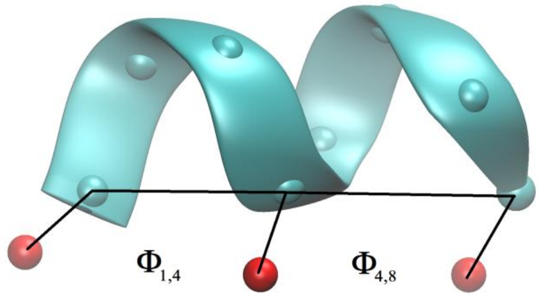


**Supplemental Figure 4.** Schematic of the improper dihedral Φ ij used to quantify the relative orientation of succssive binding groups along the length of the peptide. The helix represents the peptide backbone. The hydroxyl group oxygens (red spheres) and backbone carbons (cyan spheres) define the dihedral. Residue pairs with a dihedral value roughly ranging between ± 90° are simultaneously oriented toward the surface. Shown are two improper dihedrals. $\Phi_{\mathbf{1,4}}$ measures hydroxyl group orientation between residues 1 and 4, and $\Phi_{\mathbf{4,7}}$ provides similar information for residues 4 and 7.
